# The Promise of Artificial Intelligence in Reshaping Anticancer Drug Development

**DOI:** 10.3390/cells13201709

**Published:** 2024-10-16

**Authors:** Kostas A. Papavassiliou, Amalia A. Sofianidi, Vassiliki A. Gogou, Athanasios G. Papavassiliou

**Affiliations:** 1 First University Department of Respiratory Medicine, Medical School, ‘Sotiria’ Hospital, National and Kapodistrian University of Athens, 11527 Athens, Greece; konpapav@med.uoa.gr (K.A.P.); gogvanessa@gmail.com (V.A.G.); 2 Department of Biological Chemistry, Medical School, National and Kapodistrian University of Athens, 11527 Athens, Greece; amsof.00@gmail.com

While the concept of artificial intelligence (AI) has deep historical roots, its development as a formal scientific field was initiated in the 1950s by Newell and Simon, who invented a “thinking machine” called the Logic Theorist [1]. Since then, AI has undergone several cycles of breakthroughs and setbacks, evolving into the transformative technology we see today. Noting that the 2024 Nobel Prize in Chemistry has been awarded to work involving the application of AI models in decoding the structure of proteins and creating new ones [2], the integration of AI into anticancer drug development is revolutionizing the pharmaceutical landscape, providing novel, exciting perspectives as efficacy and toxicity can be predicted from computer-created active biomolecular structures and tumor cell signaling networks. As cancer continues to pose a significant global health challenge, the demand for more personalized and effective treatments has never been higher.

When applied to cancer, AI has the potential to substantially influence various aspects of drug development and fundamentally reform therapeutic trial planning [3]. AI can foster every stage of the drug development process, from preclinical research to clinical trial design, patient recruitment, and trial analysis [3]. By generating in silico clinical trials, AI can predict patient responses and outcomes, offering valuable insights that streamline and optimize each phase [4]. Models are generally created using data from previous trials of similar drugs, concentrating on essential parameters from a smaller set of patients. This approach also reduces the risk of duplicating an existing clinical trial design. Unlike conventional trials, in silico trials need fewer but more informative patient data, enabling them to be completed more quickly and cost-effectively [5]. A standout example of this approach is Novadiscovery’s jinkō trial simulation platform, which swiftly set up the phase III FLAURA2 trial in just one month [6], while the actual clinical trial spanned three years [7]. The platform successfully predicted that combining chemotherapy with osimertinib could remarkably enhance progression-free survival in patients with epidermal growth factor receptor (EGFR)-mutated non-small cell lung cancer (NSCLC) [6].

The average duration from the initiation of a drug discovery program to marketing approval by national regulatory agencies is approximately 12–15 years [8]. However, the recent Nobel Prize-winning invention AlphaFold, a revolutionary AI technology for predicting protein structures [9], offers tremendous promise for accelerating anticancer drug development and shortening the discovery timeline. By accurately deciphering the precise structures of proteins that cancer cells rely on for survival, AlphaFold can significantly enhance the development of drugs designed to specifically target these crucial molecules. Researchers at the National Institutes of Health (NIH) have already utilized high-resolution gene expression data from individual tumor cells to improve the predictive capabilities of an AI tool known as Personalized Single-Cell Expression-based Planning for Treatments in Oncology (PERCEPTION), allowing it to better forecast drug responses [10]. This method outperforms traditional bulk sequencing by pinpointing distinct subpopulations of tumor cells, which may respond differently to treatment regimens. The tool successfully predicted drug resistance in patients with multiple myeloma and breast cancer [10].

AI can considerably reduce health inequalities in anticancer drug development by enabling more personalized and accessible treatments [11]. By streamlining the drug discovery process, AI has the potential to lower development costs, making new drugs more affordable and widely available. Additionally, AI can create tailored treatment plans by analyzing a patient’s medical history, genetics, and lifestyle. Real-time monitoring through AI-powered devices facilitates early detection of health issues, ensuring prompt care, even for those in remote or underserved areas where access to regular healthcare may be limited. Nevertheless, the impact of AI on healthcare is a double-edged sword. While it has the potential to democratize access to care, it can also introduce biases if not carefully managed. Advanced AI technologies may not be equally accessible to all populations, and without thoughtful oversight, these disparities could deepen, leading to uneven patient outcomes [11,12,13].

In summary, the application of AI in anticancer drug development offers great hope for patients [13]. Emerging technologies like quantum computing could further strengthen AI’s role in anticancer drug discovery, potentially solving complex problems even faster [14]. However, the role of oncologists in the clinical setting remains decisive, as human ethics and judgment are essential. To fully realize AI’s potential, it is important to promote collaboration among AI experts, cancer researchers, oncologists, and regulatory bodies to establish robust frameworks for the integration of AI into drug development. This partnership can enhance the accuracy of treatments while ensuring that the human element of care is never sidelined.
